# Is There Justification to Treat Neurodegenerative Disorders by Repurposing Drugs? The Case of Alzheimer’s Disease, Lithium, and Autophagy

**DOI:** 10.3390/ijms22010189

**Published:** 2020-12-27

**Authors:** Odeya Damri, Nofar Shemesh, Galila Agam

**Affiliations:** Department of Clinical Biochemistry and Pharmacology and Psychiatry Research Unit, Faculty of Health Sciences, Ben-Gurion University of the Negev and Mental Health Center, Beer-Sheva 8461166, Israel; odeyad@post.bgu.ac.il (O.D.); shnofar@post.bgu.ac.il (N.S.)

**Keywords:** repurposing drugs, lithium, neurodegenerative disorders, Alzheimer’s disease, treatment

## Abstract

Lithium is the prototype mood-stabilizer used for acute and long-term treatment of bipolar disorder. Cumulated translational research of lithium indicated the drug’s neuroprotective characteristics and, thereby, has raised the option of repurposing it as a drug for neurodegenerative diseases. Lithium’s neuroprotective properties rely on its modulation of homeostatic mechanisms such as inflammation, mitochondrial function, oxidative stress, autophagy, and apoptosis. This myriad of intracellular responses are, possibly, consequences of the drug’s inhibition of the enzymes inositol-monophosphatase (IMPase) and glycogen-synthase-kinase (GSK)-3. Here we review lithium’s neurobiological properties as evidenced by its neurotrophic and neuroprotective properties, as well as translational studies in cells in culture, in animal models of Alzheimer’s disease (AD) and in patients, discussing the rationale for the drug’s use in the treatment of AD.

## 1. Introduction

Neurodegeneration in general relates to neuronal death in the central nervous system (CNS). Neurodegenerative diseases are characterized by gradual anatomical and/or physiological aberration of neuronal systems. They include Huntington’s disease (HD), Alzheimer’s disease (AD), amyotrophic lateral sclerosis (ALS) and Parkinson’s disease (PD) [1]. As of now-a-days all neurodegenerative diseases are clinically unmanageable. This situation might get worse as the aging population is increasing worldwide. Here we briefly review the different types of neurodegenerative diseases and successful or unsuccessful treatment avenues with emphasis on AD. In particular, we review and discuss pros and cons to repurpose lithium salts (lithium), the prototype drug for bipolar disorder, for the treatment of AD.

### 1.1. Huntington’s Disease

Huntington’s disease (HD), an inherited autosomal disease [2], is characterized by ‘intraneuronal nuclear inclusions’ (INNs)] composed of insoluble aggregates of extra-long polyglutamine caused by a gain-of-function mutation in the huntingtin (*HTT*) gene [3,4] causing expansion of cytosine–adenine–guanine (CAG) repeats at the N-terminus of the protein. The accumulation of the nuclear inclusions results in progressive cortical and striatal neuronal loss and a disease characterized by gradual progress from motor disturbances, cognitive decline, bradykinesia, rigidity, muscle wasting, weight loss and, eventually, death [2]. Mechanisms including mitochondrial dysfunction, oxidative stress and apoptosis have been implicated in the pathology of HD [3]. Nevertheless, there are currently no treatment options for HD.

### 1.2. Parkinson’s Disease

Parkinson’s disease (PD) is characterized, clinically, by muscular rigidity, tremors and bradykinesia, and, pathologically, by progressive loss of dopaminergic neurons in the *substantia nigra pars compacta* (SNpc) and, later, by mutant protein accumulation in Lewy bodies [5]. Two proteins are involved in the pathophysiology of PD—α-synuclein, implied as a regulator of dopamine neurotransmission [6]. It is normally translocated into lysosomes for degradation by the chaperone-mediated autophagy (CMA) pathway [7] and Park2, coding for the ubiquitin-ligase parkin. In mammalian cells parkin is recruited to dysfunctional mitochondria with reduced membrane potential, causing mitochondria engulfment by autophagosomes. In familial PD pathogenesis involves accumulation of mutant α-synuclein in the Lewy bodies [8,9]. Mutated α-synuclein binds to the lamp2a receptor located on the lysosomal membrane and, as such, inhibits their own degradation and that of other substrates. As a compensatory response, the latter results in autophagy upregulation [10]. Furthermore, failure to eliminate dysfunctional mitochondria has been reported in Park2 mutants [11]. Together, these studies strongly suggest the involvement of impaired autophagy and mitochondrial dysfunction in the pathophysiology of PD. Although up until now there is no drug to reverse the degeneration of striatal neurons, there do exist effective treatments to ease PD symptoms.

### 1.3. Multiple Sclerosis (MS)

MS is a chronic inflammatory disease of the CNS with an unknown etiology, but both genetic factors related to immune function and activation [12] and environmental factors such as Epstein-Barr virus infections [13] are believed to be involved in its pathophysiology. The patients exhibit focal plaques of demyelination in the spinal cord as well as in the white and the grey brain matter [14]. There are several types of the disorder: (I) In most of the patients the disease begins with a course of ‘relapsing-remitting’ (RRMS). After several years it is followed by a ‘secondary progressive’ phase (SPMS). Patients with ‘primary progressive’ disease (PPMS) miss the relapsing-remitting stage. They demonstrate uninterrupted progression from disease onset [15]. (II) Acute MS-patients die within the first year of the disease [16]. Some evidence suggests that entrance of autoreactive T lymphocytes from the periphery to the CNS initiates lesion formation [17] and, hence, that the disease is an autoimmune one. The latter supposition is supported by the fact that aggressive immunomodulatory treatments reduce disability progression and relapses [18]. Trapp and Nave suggested that MS primarily occurs due to either brain infection or neuronal disturbance, and that inflammation represents a secondary response [19]. There is currently no cure for MS, but a variety of drugs exists for speeding recovery from attacks, slowing progression and managing symptoms. These are beyond the scope of the present review.

### 1.4. Amyotrophic Lateral Sclerosis (ALS)

ALS is a fatal progressive disorder. It starts focally, affecting motor neurons and results in muscle weakness of the limbs and the respiratory system [20]. Its prevalence is ~5/100,000 cases/year [21]. About 90% of cases are sporadic (sALS) and 10% familial (fALS) [22,23]. Both types can develop concurrently with frontotemporal lobar dementia (FTLD). FTLD patients exhibit behavioral changes, progressive aphasia and, sometimes, movement disorders [24,25]. Differently from AD which involves hippocampal pathology, in FTLD the early atrophy resides in the frontal and temporal lobes. As for the treatment of ALS, success obtained in laboratory models failed so far in human clinical trials.

### 1.5. Alzheimer’s Disease (AD)

AD is phenotypically characterized by memory loss and progressive cognitive decline [26] and pathologically—by the presence of senile plaques. The plaques are produced by proteolysis of the amyloid-precursor protein (APP) [27]. It is assumed that the accumulation of abnormal proteins like hyperphosphorylated Tau is the main component of neurofibrillary tangles [28]; accumulation of amyloid beta (Aβ) aggregates is responsible for the senile plaques while α-synuclein can aggregate to form pathological Lewy bodies [29], induce neuronal loss and further neuronal network dysfunction.

There are currently six Food and Drug Administration (FDA)-approved drugs that somewhat temporarily slow disease progression [30], but the actual fact is that a treatment to reverse or, at least, to arrest deterioration is still unavailable. The approved treatments are based on two strategies: symptomatic-cholinesterase inhibitors and the non-competitive NMDA receptor antagonist memantine approved for use in moderate to severe AD, and disease modifying treatments—antioxidants and anti-inflammatory agents. All are just palliative, temporarily slowing cognitive decline and prescribed due to lack of a better alternative. Hope for immunotherapy with antibodies against Aβ plaques and against fibrillary tangles encounter, in humans, side effects of significant concern. Several recent clinical trials based on anti-amyloid or anti-Tau failed. What if several toxic agents act concomitantly causing neuronal death? Then it might turn out that blocking all these agents will be required to prevent or delay neuronal death. In this respect it is of interest to note that (i) abnormal increase in GSK-3 levels and activity have been reported to be associated with brain pathogenesis (neuronal death) in AD patients [31,32], (ii) GSK-3 has been shown to acts as a regulator of Aβ production [33,34,35] as well as a tau kinase [36,37,38], and (iii) the mood stabilizer lithium has been shown to inhibit GSK-3 *in vitro.*

### 1.6. Autophagy and AD

In neurons, autophagy is a crucial regulator of cellular homeostasis, the balance between synthesis, degradation and recycling of cellular components, and in synapse plasticity required for learning and memory that is impaired in AD [39,40,41]. Yet, despite studies in cells in vitro, in animal models and in AD patients showing a direct link to aberrant autophagy, the specific mechanism underlying autophagy dysfunction in AD is not fully understood [42]. It has been shown that in AD autophagic vacuoles accumulate in parts of neurons, possibly due to impaired autophagy vacuoles clearance [43], but may also reflect enhanced autophagy induction or defective later lysosomal degradation process [44]. A recent study [45] demonstrated that enhanced mitophagy (elimination of defective mitochondria, a subtype of macroautophagy) abolishes AD-related tau hyper-phosphorylation in vitro and reverses memory impairment in transgenic tau mice in vivo, suggesting that impaired removal of defective mitochondria is a key factor in AD pathogenesis. These studies and similar ones point at autophagy mechanisms as potential therapeutic interventions in AD. Lithium is a well-known autophagy enhancer through inhibition of IMPase1 and induction of the mTOR-independent mechanism of autophagy enhancement [46]. Hence, lithium’s potential therapeutic use in treating AD should not be ruled out. Henceforth, our aim was to review lithium’s neurobiological, neurotrophic, and neuroprotective properties and to discuss the rationale for its repurposed use in the treatment of AD.

## 2. Methods

The strategy used to prepare this review was to search relevant manuscripts from electronic databases such as PubMed and Google Scholar published in English between 1947 and 2020. Reference lists of articles were used to enrich the review. Search terms included lithium with the AND operator to combine with mechanism, OR with Alzheimer’s. The latter were then subdivided into cellular models, animal models and patients. Data extracted from the included papers was initially used by the first author to collate it in a Microsoft Word document summarizing general characteristics and intervention design in a structured manner. The second author verified the data. The corresponding author resolved discrepancies and wrapped the review.

### 2.1. Repurposing Lithium as an AD Drug Development Strategy

By repurposing we mean using a drug or a candidate drug for a disease different from the original one for which it was primarily intended [47]. The new use may intend targeting the original mechanism of action or a new one, e.g., extend the indication range from mild/moderate state to also include a severe stage. By using existing pharmacokinetic and safety data drug repurposing may reduce risk associated with new drug discovery. It has been proposed that for AD drug repurposing might be an attractive strategy [48,49,50,51]. Indeed, in the case of neurodegenerative disorders, in general, and in AD, in particular, application of already approved therapies as repurposed treatment means has been loomed both at the basic science and at the clinical level [48]. One of the most promising repurposing drug for AD is lithium, given the drug’s neurotrophic and neuroprotective properties [49,52,53,54].

### 2.2. Why Lithium?

Lithium was discovered by the Swedish chemistry student Arfwedson in 1817. In the mid-19th century physicians observed that lithium dissolved kidney uric acid in vitro and studied its potential treatment for gout. In 1949 Cade discovered that lithium induces sedation in guinea pigs [55]. He hypothesized that mental illnesses result from intoxication by an unknown compound that might be present in the patients’ urine. Since lithium urate is the only soluble salt of uric acid he injected it into guinea pigs. Noticing that the guinea pigs became sedated drove him to treat psychiatric patients with lithium carbonate. Patients with either schizophrenia or depression did not improve but in all ten mania patients symptoms ceased or were reduced [55]. This led to the introduction of lithium as an anti-manic drug, its efficacy still being reported to be the strongest [56]. Further studies by Schou’s group substantiated lithium’s therapeutic and prophylactic benefit [57,58]. Lithium became an FDA approved drug for BD maintenance in 1974.

### 2.3. Neuroprotective Effects of Lithium

Accumulated data in the past decades implies that lithium’s neurobiological benefits expand beyond mood stabilization. Lithium’s neuroprotection characteristics resides from its involvement in mechanisms of neuronal homeostasis. In particular, in-vivo and in-vitro studies demonstrated that, as a neuroprotective agent, lithium hampers apoptosis-induced cell death [59,60,61], modulates oxidative stress [62,63,64,65] and inflammatory cascades [66] and up-regulates mitochondrial function [64,67,68] and autophagy [46,69]. All latter responses are, possibly, secondary to the inhibition of two key enzymes—glycogen synthase kinase (GSK)-3β and inositol-monophosphatase (IMPase) [70].

There are two mammalian isoforms of GSK-3-alpha and -beta, encoded by independent genes. The isoforms share similar kinase catalytic domains, they function identically in Wnt signaling, but differ in the rest of their structure, substantially in their termini [71,72], in tissue distribution and in some metabolic roles as follows. The β isoform is more abundant in brain. It is implicated to be involved in organization and remodeling of the cytoskeleton [73]. Conversely, cerebral GSK-3α is involved in neurodevelopment [74]. Interestingly, using a transgenic mouse model of AD, lithium inhibition of GSK-3α has been related to disease-modification [75], but Su et al. [34] documented that inhibition of GSK-3β also results in Aβ formation from APP. Some groups believe that lithium’s inhibition of GSK-3β substantiates its potential as a repurposing drug in the treatment and/or prevention of AD [76,77]. There are two ways by which lithium inhibits GSK-3β activity: (a) directly, by competing with the binding of Mg^2+^ ions to the catalytic site [78,79]; (b) indirectly, through inducing phosphorylation of the serine-9 residue of GSK-3β resulting in the enzyme’s inactivation. Downstream of the indirect cascade intracellular kinases (Akt) are activated while intracellular protein phosphatase 2A (PP2A) is inhibited [59,80,81]. In addition, lithium has been shown to inhibit mRNA transcription of GSK-3β, thus reducing the enzyme’s availability [82].

Another mechanism of action of lithium is the inhibition of IMPase [83] and inositol polyphosphate-1-phosphatase (IPPase) [84]. As in the case of GSK-3β, lithium directly inhibits IMPase and IPPase activity by interfering with the presence of Mg^2+^ ions in the enzyme’s catalytic site [85]. Inhibition of IMPase and IPPase prevents the recycling and release of free inositol, thus depleting inositol’s intracellular levels attenuating signaling via the phosphatidylinositol (PI) cascade mediated by the two second messengers inositol triphosphate (IP_3_) and diacylglycerol (DAG) [86]. IP_3_ and DAG regulate intracellular pathways relevant to neuropsychiatric disorders, among which is the autophagy process [87].

It is noteworthy that manifestation of different neurodegenerative diseases such as AD [88,89], ALS [90,91], Parkinson’s [92,93], and Huntington’s disease [94,95,96] share similar pathogenesis characteristics including neuroinflammation, mitochondrial dysfunction, enhanced apoptosis and attenuated autophagy—all positively affected by lithium. Indeed, studies of the potential beneficial effect of lithium in neurodegenerative disorders, tested at the cellular, animal models and patients levels are cumulating.

Below we review those reported in AD.

### 2.4. Evidence for Neuroprotective Effects of Lithium in Cellular Models of AD

Paired helical filaments (PHFs, of which Tau is the main component) are brain anomalous structures found in patients with tauopathies such as AD and FTDP-17. Go’mez-Ramos et al. [97] developed an in-vitro model which favors the formation of tau aggregates. They infected *Spodoptera frugiperda* (Sf)9 insect cells (an established cell line for infection with recombinant baculovirus) with baculoviruses containing the wild type (tau ^42^) and an altered form of tau (tau^VLW^, characteristic of FTDP-17) to study whether tau aggregates are formed. The results supported the formation of tau filaments resembling PHFs and behaving biochemically as a hyperphosphorylated form of the protein. Using this paradigm, they found that lithium treatment decreases the formation of the tau filaments. They concluded that the blockage of GSK-3 in tau^VLW^-expressing cells affects the capacity of this altered form of tau to assemble into filamentous polymers [97]. A study by Sun et al. [98] examined the effect of lithium (for 12 or 24 h at concentrations of 0, 5, 10, and 20 mM) on Aβ secretion from COS7 cells, which express lytic SV40 large T antigen, transfected with APP C100. Lithium treatment reduced the secretion of both Aβ40 and Aβ42 in a dose-dependent manner. They also determined that diminished amyloid secretion was associated with GSK-3β activity. The authors concluded that “lithium should be re-evaluated as a candidate reagent for preventing AD pathology” [98].

Acetylcholinesterase (AChE) hydrolyzes and inactivates acetylcholine (ACh) at cholinergic synapses and neuromuscular junctions [99]. Its activity is increased in the process of amyloid deposition in plaques and tangles [100]. Recent years’ evidence suggests the possible involvement of synaptic AChE (AChE-S) in apoptosis [101,102]. Therefore, Jing and colleagues [103] hypothesized that the loss of neurons during AD development may be due to neuronal cell apoptosis and that AChE-S might play an important role in AD. They demonstrated that GSK-3β could increase AChE-S protein stability. Confocal microscopy experiments showed that GSK-3β and AChE co-localized extensively in the cytosol of PC12 cells. Therefore, they hypothesized that GSK-3β may regulate AChE. However, PC12 cells express two AChE isoforms: AChE-S and readthrough acetylcholinesterase (AChE-R), thereby making it difficult to determine the exact relationship between GSK-3β and AChE-S in these cells. Thus, they examined the levels of ectopically expressed AChE-S in HEK293T cells treated with 20 mM LiCl for 24 h (note that 20 mM LiCl is way above therapeutically-relevant levels). The results indicated that lithium treatment led to a significant downregulation of AChE-S activity and protein expression. However, the destabilizing effect of lithium on AChE-S was inhibited by the proteasome inhibitor MG132, suggesting that lithium treatment led to rapid proteasomal degradation of AChE-S. Lithium-mediated AChE-S degradation could be reversed when a constitutive active form of GSK-3β (Ser9Ala) was co-transfected with AChE-S. Their results suggest that AChE-S stabilization was GSK-3β-dependent [103].

De Ferrari and colleagues reported that Aβ in neuronal cells in culture formed Aβ fibrils [104]. Considering that lithium mimics Wnt signaling (essential in developmental and oncogenic processes) by reversibly inactivating GSK-3β [78,105] they examined whether lithium demonstrates neuroprotective action against Aβ-dependent damage. Since others’ previous studies suggested that Wnt signaling inactivates GSK-3β via protein kinase C (PKC) [106,107,108], they also investigated whether PKC agonists/inhibitors modulate Aβ-induced neurotoxicity. It turned out that Aβ fibrils destabilize β-catenin’s intracellular levels (β-catenin is a key transducer of Wnt signaling). Inhibition of GSK-3β activity either by lithium, by the PKC activator phorbol-12-myristate 13-acetate (PMA), or by conditioned medium containing the Wnt-3a ligand, all of which activate Wnt signaling, protected post-mitotic neurons from Aβ-induced neurotoxicity [104]. In a similar manner, Rametti et al. [109] found that exposure of cultured cortical neurons to lithium reduced tau protein and mRNA levels, supporting the possibility that lithium’s neuroprotective effect against Aβ toxicity is mediated by reduction of tau mRNA. This corroborates reports that lithium modulates transcription factors of Wnt signaling and, thereby, target genes expression [110]. Lithium treatment has also been reported to decrease tau phosphorylation facilitating tau destruction [111].

Despite some inconsistencies, by-and-large, the studies using AD cellular models support the notion that repurposing lithium for the treatment of AD patients should not be ruled out.

### 2.5. Evidence for Neuroprotective Effects of Lithium in Animal Models of AD

As mentioned above, the Aβ is produced by sequential secretase-dependent proteolytic processing of APP and chronic lithium treatment is robustly reported to block Aβ production. For example, Phiel et al. demonstrated that chronic lithium treatment blocked Aβ accumulation in CHO-APP695 cells and primary mice neurons [75]. A different mechanism was suggested using mice that underwent traumatic brain injury (TBI). In this model lithium attenuated APP processing through brain inhibition of β-secretase-1 (BACE-1) [112], an effect mimicked in-vitro by transfection with siRNA of GSK-3α but not of GSK-3β [75] by showing that GSK-3β inhibition mimics lithium’s capability to suppress Aβ formation from APP [34]. A novel mechanism, by which GSK-3 directly regulates Aβ42 levels along with no effect on APP processing has been demonstrated in a study in a Drosophila model of AD induced at adulthood [113,114]. Moreover, treating transgenic mice overexpressing human APP with lithium for three months diminished both Aβ levels and tau hyper-phosphorylation, avoided cortical and hippocampal neurodegeneration and alleviated memory deficits [35]. In transgenic mouse models of AD, such as mice expressing mutant tau, chronic lithium treatment reduced aggregation of the mutant protein [115], detained neurofibrillary tangles development [116], as well as boosted ubiquitination rendering decreased tau-induced lesions [117]. Caccamo et al. [118] administered lithium to 15-months-old homozygous 3xTg-AD mice that harbor both plaques and tangles vs. non-Tg mice for four weeks. Lithium reduced tau phosphorylation but had no effect on the Aβ load and no improvement of deficits in working memory [118], inconsistent with the report that long-term lithium treatment of rats injected with Aβ fibrils amended the animals’ deficits in spatial learning [104]. Tau phosphorylation may be regulated by both GSK-3 and by protein phosphatase 2A (PP2A) [119]. Lithium treatment has been reported to increase rat brain PP2A activity [120].

Trujillo-Estrada and colleagues [121] treated the PS1xAPP transgenic mouse model of AD with lithium and report rescue of memory impairment, reduced Aβ plaque toxicity, lack of hippocampal and entorhinal cortex neuronal loss, lower phospho-tau accumulation, reduced steady-state levels of LC3-II and less ubiquitinated proteins. They raise the possibility that lithium’s effects could have been mediated by astrocyte activation and release of heat shock proteins concentrating in the core of the Aβ plaques. However, in another APP transgenic mouse model, Sudduth et al. [122] found that lithium treatment for eight months, despite significantly lowering hyperphosphorylated tau levels, did not induce neuroprotection and did not affect spatial memory, increased amyloid deposition did occur, Aβ levels were unaltered and brain’s normal neuroinflammation phenotype was significantly altered. Investigating the effects of chronic lithium treatment in aged double transgenic mice (AβPP/PS1) Zhang and colleagues [123] found that lithium reduced the γ cleavage of APP, decreased Aβ production and senile plaque formation, improved spatial learning and memory and attenuated autophagy activation. Later on Pan et al. showed [124] that chronic lithium treatment (300 mg/kg/day for 21 days) of the same APP/PS1 mouse model exhibited a 31% increase in the brain clearance of Aβ42. The clearance was associated with an increase in brain microvascular expression of the blood-brain- barrier efflux transporter low density lipoprotein receptor-related protein 1 (LRP1) and cerebrospinal fluid (CSF) bulk-flow, suggesting an alternative lithium-induced protection mechanism. Finally, using 100 mM lithium, a way beyond therapeutically-relevant dose, Nery et al. [125] showed that the drug ameliorated cognitive deficits and increased tau phosphorylation in GSK-3β target residues in zebrafish injected with Aβ_1–42_ into the hindbrain ventricle of embryos.

An intriguing novel approach to study the potential beneficial effect of lithium in AD-like animal models is using lithium microdoses. Towards this goal Nunes et al. [126] studied the effect of chronic microdose lithium treatment (0.25 mg/kg/day in drinking water) administered either before or after the appearance of symptoms used transgenic mice modeling a disease similar to AD. They treated mice of two or ten months of age for eight or 16 months, respectively, with either lithium carbonate in drinking water or water only. Unlike the water-treated transgenic mice neither the lithium-treated transgenic groups nor the non-transgenic mice showed memory disruption at the end of the treatment. Furthermore, Liu et al. [127] recently reported that long-term administration of low-dose lithium (both 5 and 17.5 mg/kg/day LiCl) significantly improved spatial memory of APP/PS1 mice and reduced Aβ plagues and p-tau levels. Zhao et al. [128] designed and synthesized tri-lithium pyrroloquinoline quinone (Li_3_PQQ). Li_3_PQQ is a naturally occurring redox cofactor with powerful antioxidant action and mitochondrial amelioration. In APP/PS1 transgenic mice a relatively low dose of the new compound restored impaired learning and memory, facilitated hippocampal long-term potentiation (LTP) and reduced cerebral amyloid deposition and phosphorylated tau level significantly more than low or high lithium chloride doses. The authors also report that Li3PQQ inhibited GSK-3 activity and increased β-amyloid-binding alcohol dehydrogenase (ABAD) activity, a mitochondrial energy regulator protein. The latter effects might have mediated Li3PQQ’s beneficial effects. Similarly, Medesis Pharma (Montpellier, France) developed NP03, another novel microdose lithium formulation which allows enhanced CNS uptake by transmucosal administration, resulting in blood lithium concentrations below detection limit but high bioavailability [129]. Using this formulation to treat McGill AD transgenic rats Cuello’s group demonstrated it halted evolvement of AD-like pathology [130]. Namely, it rescued early learning and memory deficits via restoring CRTC1 (transcriptional coactivator for CREB1; CREB = cyclic AMP responsive element binding protein 1) promoter occupancy in synaptic plasticity genes, induced an inactivated GSK-3β profile, re-established hippocampal neurogenesis in the AD-like rats and dampened *Bace1* (β-site APP cleaving enzyme-1) gene expression and activity thereby reducing Aβ42 levels [131]. Recently (2020), using the same AD-like transgenic rats, the group reported that administration of NPO3 during the early Aβ post-plaque stage rescued AD-related functional and molecular biomarkers. Namely, it restored object recognition and reduced loss of hippocampal cholinergic boutons and Aβ plaque number, soluble and insoluble cortical Aβ42 levels, markers of neuroinflammation and cellular oxidative stress [132]. The results indicate that microdose lithium in the NP03 formulation is effective in reversing key AD-like pathologies in an in-vivo model both in the early stages of the disease and at later stages, after appearance of Aβ plaques. It is of interest to note that the Ki for lithium inhibition of GSK-3 and IMPase is ~2 and ~1 mM, respectively. Blood lithium concentration in bipolar patients on lithium treatment is at the range of 0.7–1.2 mM, corroborating with the hypothesis that the drug’s beneficial effect is mediated via inhibition of one of these enzymes or both. This situation is not plausible in the case of lithium microdose. Therefore, the mechanism of the above effects remains to be investigated.

Although the animal models results are not in full agreement, clinical trials were also carried out.

### 2.6. Clinical Evidence for Neuroprotective Effects of Lithium in AD Patients

As described above, preclinical models of AD demonstrated that lithium can attenuate or even eliminate Aβ and tau pathology along with improvement of cognitive function. Based on these findings few clinical studies were carried out in AD patients. In a retrospective study of patients treated chronically with lithium Terao et al. [133] found that lithium-treated patients exhibited significantly better cognition and memory compared with those on other treatments. Similarly, Kessing et al. found that continuous lithium treatment reduced dementia rate or AD [134]. In a clinical trial carried out by Nunes et al. [135] chronic treatment of elderly bipolar patients with lithium resulted in significantly lower AD incidence (7 vs. 19%) compared to patients who were not exposed or minimally exposed to lithium during their lifetime. In contrast, MacDonald et al. [136] found no significant effect of lithium on cognitive function after a year of treatment of mild to moderate AD patients. Yet, the safety of lithium in old adults was deduced from the mild and reversible side effects [136]. Hampel et al. [137] also did not find a beneficial effect of lithium on cognition following 10 weeks of treatment at therapeutically-relevant levels (0.5–0.8 mmol/L). They also did not find changes in CSF levels of Aβ42 and leukocytes levels of phosphorylated tau and phosphorylated GSK-3β—regarded as AD biomarkers. However, plasma BDNF (brain-derived neurotrophic factor) levels but not those of CSF and serum glial cell-derived neurotrophic factor (GDNF) were increased. Furthermore, in a subgroup of the participants in whom baseline BDNF levels were low, lithium treatment restored them to be similar to controls, and cognitive parameters of these patients significantly improved [138]. This is reminiscent of the Hashimoto et al.’s report that BDNF induction by lithium was necessary for the protection against glutamate-induced apoptotic death of primary cortical neurons [139], and that lithium-induced BDNF involved inhibition of both GSK-3α and GSK-3β isoforms in primary cortical neurons [140]. A meta-analysis of three randomized placebo-controlled clinical trials including 232 participants showed beneficial effects of lithium treatment on cognitive performance in subjects with mild cognitive impairment (MCI) and AD dementia [141].

Aiming to explore if long-term sub-therapeutic lithium levels (<0.5 mmol/L serum) delay the progression of AD cognitive symptoms from mild amnesia to dementia, Forlenza et al. [77] carried out a double-blind, placebo controlled clinical trial. Following a year of lithium treatment amnestic subjects demonstrated stable cognitive characteristics, a similar conversion rate to AD and significantly reduced CSF levels of phosphorylated tau as compared with subjects on placebo. These results support the notion that long-term lithium exerts a beneficial effect on core biological features of AD with subtle clinical benefit, in particular when started at early disease stages. Following the rationale of using lithium microdoses to reduce toxicity risk, Nunes et al. [142] found that 15 months of minute lithium doses prevented the decrease in performance found in the placebo group in the mini-mental state examination test starting at three months of treatment and increasing progressively. As discussed under the subtitle ‘Evidence for neuroprotective effects of lithium in animal models of AD’ the mechanism by which lithium microdoses exert their reported beneficial effects in AD remains to be unraveled. We contend that the above reviewed data reinforce the therapeutic potential of lithium (either micro- or standard-dose) in preventing cognitive loss in AD.

Some studies report that brain abnormalities in AD patients are reflected in peripheral cells. One such finding is decreased fibroblast PKC levels [143]. Based on reports that lithium affects PKC function [144,145] Molchan et al. reported the effect of three weeks of lithium treatment on platelet protein levels of four PKC isozymes in patients with probable AD as compared with age-matched controls. Membrane-associated PKC ζ levels were found significantly lower in the AD patients both prior and following lithium treatment [146]. The authors interpret that some peripheral cells’ PKC isozymes are deregulated in AD and that lithium treatment modifies their regulation.

### 2.7. Lithium Toxicity

In bipolar disorder and other maladies in which lithium treatment is in use the drug’s toxicity is not uncommon due to its narrow therapeutic index. Clinical manifestations of lithium toxicity include renal dysfunction, neurologic dysfunction, gastrointestinal upset, cardiac manifestations and endocrine abnormalities. We hereby shortly relate to each of them.

Renal dysfunction—lithium can induce nephrogenic diabetes insipidus (NDI) in up to 87% of the patients [147], but its ability to cause chronic kidney disease (CKD) and further end stage kidney disease (ESKD) appears to be very low. Aprahamian et al. reported that four years of lithium treatment at sub-therapeutic doses had no significant changes in renal function in elderly adults [148]. Moreover, development of ESKD is associated with a significant latent period. Namely, significant nephrotoxicity appears following more than 25 years of treatment [149].

Neurologic dysfunction—lithium-induced toxicity primary site is the CNS. The range of the clinical indices is from asymptomatic, through confusion, ataxia and up to seizures. Lithium poisoning has a low mortality rate and persistent cerebellar neurological deficits are uncommon in uncomplicated acute poisoning [150].

Gastrointestinal upset, including gastrointestinal pain or discomfort and diarrhea, are adverse effects of lithium which are usually minor and often transient [151].

Cardiac manifestations—toxic lithium levels may result in cardiovascular effects ranging from relatively benign ST-T wave changes to fatal arrhythmias [152].

Endocrine abnormalities—the most prominent endocrine effect of lithium treatment relates to the drug’s influence on the thyroid gland, a key side effect in lithium’s long-term therapy. Lithium administration leads to reduced production and/or inhibited release of thyroid hormones, resulting in goiter and hypothyroidism. A rare complication is hyperthyroidism [153].

The state of the art now-a-days is that none of the drugs administered to Alzheimer’s patients “deliver the goods”. Hence, thinking ‘out of the box’ is mandatory. Although, as summarized above, lithium is a drug with serious side effects in old patients in particular, this is correct under two conditions: (a) if a subject is exposed to an over-dose; (b) if a subject is treated for more than two decades. Both conditions are not expected to occur in Alzheimer’s patients: (a) Most of the patients are looked after by care-givers, so overdose is expected to happen rarely. Hence, if blood lithium levels are carefully monitored the chance of lithium toxicity is minimized and the major concern remains renal dysfunction. (b1) Given the age of onset of AD, the life expectancy of most of the patients does not exceed 25 years, when kidney disease might outburst. Therefor, the chance for renal dysfunction is also minute. (b2) We contend that if given the choice between becoming demented or requiring dialysis, most patients will choose the latter. Two reviews, each by a prominent psychiatrist, one from Europe and one from the US, both with decades of clinical and translational science experience, advocate the use of lithium treatment despite its side effects [154,155].

## 3. Conclusions

In this review we wished to raise the question whether there is justification to treat AD disease by repurposing lithium, the prototype mood-stabilizer used for acute and long-term treatment of bipolar disorder. We first reviewed translational research which indicates the drug’s neuroprotective characteristics, namely, its neurobiological properties as evidenced by its neurotrophic and neuroprotective effects culminating in modulation of homeostatic mechanisms, such as inflammation, mitochondrial function, oxidative stress, autophagy and apoptosis. We summarized that the myriad of intracellular responses is, possibly, a consequence of the drug’s inhibition of the enzymes IMPase and GSK-3. We further reviewed translational studies in cells in culture, in animal models of AD and in patients, discussing the rationale for the drug’s use in the treatment of AD. As summarized in Table 1, manifold pieces of evidence originating from preclinical models and clinical studies are in favor of further exploring the neuroprotective potential of lithium in AD. As reviewed above, this characteristic of lithium stems from the drug’s modulation of multiple processes and pathways involved in neuronal plasticity, cell survival, energy-related metabolic activity, transcriptional control, and resistance against neurotoxic insults. Some of these mechanisms are core pathological processes of AD. Taken together, the reviewed results advocate that studying longer treatment periods in larger groups of patients are required to firmly reveal or rule out the potential benefits of lithium in these patients.

## Figures and Tables

**Table 1:** Summary of findings of the effect of lithium on AD and its models.

	Findings	Lithium Levels	References
**Cells in culture**	↓ Tau filaments, protein and mRNA levels. ↓ Amyloid secretion. ↓ AChE-S protein levels and activity via GSK3 inhibition.	2.5–20 mM	[97,109] [98] [103]
**Animal models**	↓ Tau phosphorylation and mutant. ↓ Aggregation Aβ production and accumulation. ↓ Neuronal degeneration. ↑ Ubiquitination in the brains Improved spatial learning & memory. ↑ Autophagy activation.	0.18, 0.6, 5, 10, 15, 17, 20 mM 0.02–1.2 g/kg	[35,75,111,118,121,122,127,156] [34,75,111,112,117,121,123,124,127] [156] [117] [123] [123]
**Patients**	↔ Cognitive function, Aβ42 and CSF phosphorylated tau levels. ↓ Stabilized cognition and memory, CSF phosphorylated tau levels.	Serum levels 0.5–1.5 mM	[133,136,137,138] [77,134,135,138,157]

↓ denotes reduction. ↑ denotes incease. ↔ denotes lack of change.

## Abbreviations

IMPase	inositol-monophosphatase
IPPase	inositol polyphosphate-1-phosphatase
GSK-3	glycogen-synthase-kinase-3
AD	Alzheimer’s disease
CNS	central nervous system
HD	Huntington’s disease
ALS	amyotrophic lateral sclerosis
sALS	sporadic ALS
fALS	familial ALS
FTLD	frontotemporal lobar dementia
PD	Parkinson’s disease
INNs	intraneuronal nuclear inclusions
HTT	huntingtin gene
CAG	cytosine–adenine–guanine
SNpc	substantia nigra pars compacta
LBs	Lewy bodies
CMA	chaperone-mediated autophagy
MS	Multiple sclerosis
RRMS	relapsing-remitting
SPMS	secondary progressive phase
PPMS	primary progressive disease
APP	amyloid-precursor protein
Aβ	amyloid beta
FDA	food and drug administration
IP3	inositol triphosphate
DAG	diacylglycerol
PHFs	paired helical filaments
Ach	acetylcholine
AChE	acetylcholinesterase
AChE-S	synaptic AChE
AChE-R	readthrough acetylcholinesterase
TBI	traumatic brain injury
BACE-1	β-secretase-1
PP2A	protein phosphatase 2A
PS1	presenilin-1
LiPQQ	tri-lithium pyrroloquinoline quinone
ABAD	β-amyloid-binding alcohol dehydrogenase
CREB1	cyclic AMP responsive element binding protein 1
CRTC1	transcriptional coactivator for CREB1
Bace1	β-site APP cleaving enzyme-1
CSF	cerebrospinal fluid
BDNF	brain-derived neurotrophic factor
GDNF	glial cell-derived neurotrophic factor
PKC	protein kinase C
NDI	nephrogenic diabetes insipidus
CKD	chronic kidney disease
ESKD	end-stage kidney disease
LRP1	lipoprotein receptor-related protein 1
